# Differential expression of phosphoinositide 3-kinase/protein kinase B and Toll-like receptor/nuclear factor kappa B signaling pathways in experimental obesity Wistar rat model

**DOI:** 10.1515/biol-2022-1014

**Published:** 2024-12-31

**Authors:** Liya Song, Lihua Li

**Affiliations:** Department of Children’s Center, Beijing Luhe Hospital Affiliated to Capital Medical University, Beijing, 101149, China

**Keywords:** obesity rat model, high-fat diet, phosphatidylinositol-3-kinase/protein kinase B, Toll-like receptor/nuclear factor kappa B, differences, expression

## Abstract

This study aimed to investigate the differential expression of the phosphoinositide 3-kinase (PI3K)/protein kinase B (AKT) signaling pathway in relation to the Toll-like receptor (TLR)/nuclear factor κB (NF-κB) signaling pathway in an obese rat model. A total of 200 8-week-old male Wistar rats were randomly assigned to a control group (Ctrl, *n* = 40) and an observation group (Obs, *n* = 160), with obesity induced through a high-fat diet. Following modeling, the Obs group was further divided into a model group, a PI3K/AKT inhibition group, a TLR/NF-κB inhibition group, and a combined PI3K/AKT + TLR/NF-κB inhibition group, with 40 rats in each. Metabolic changes were assessed by monitoring the glucose infusion rate (GIR), as well as conducting an intraperitoneal glucose tolerance test (IPGTT) and an intraperitoneal insulin tolerance test (IPITT). Hematoxylin and eosin staining was utilized to observe morphological changes in adipose tissue, while Western blotting was employed to detect the expression levels of proteins associated with the PI3K/AKT and TLR/NF-κB signaling pathways in adipose tissue. The results indicated that the Obs group exhibited significantly higher blood glucose and insulin levels during the IPGTT and IPITT experiments compared to the Ctrl group (*P* < 0.05). Additionally, the GIR, as well as the expression levels of p-PI3K and p-AKT proteins in the Obs group, were significantly lower than those in the Ctrl group (*P* < 0.05). In both the PI3K/AKT inhibition group and the combined PI3K/AKT + TLR/NF-κB inhibition group, the expression of relevant proteins further declined (*P* < 0.05). These findings suggest that while a high-fat diet decreases the activity of the PI3K/AKT signaling pathway, it concurrently promotes inflammatory responses by upregulating the TLR-4 and NF-κB signaling pathways, indicating a critical role for these pathways in obesity-related metabolic abnormalities.

## Graphical abstract

This study investigated the effects of activation and inhibition of the PI3K/AKT and TLR-4/NF-κB signaling pathways on insulin resistance in a high-fat diet-induced obese rat model. The results indicate that a high-fat diet can activate these signaling pathways by regulating the expression of related proteins, affecting metabolic health. Rat PI3K/AKT pathway protein.

**Figure:**
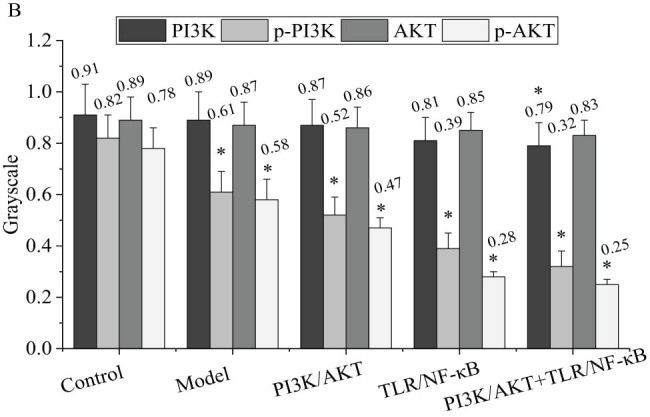

## 1 Introduction

In recent years, the accelerated process of urbanization globally has led to significant changes in lifestyle and dietary habits, resulting in obesity becoming a global public health issue, with a particularly sharp increase in obesity rates in China [1,2,3]. Obesity is not only a major contributor to various metabolic diseases but is also closely associated with a range of health issues, including cardiovascular diseases, diabetes, and fatty liver. Epidemiological studies indicate a significant association between obesity and type 2 diabetes. In obese states, adipocytes in adipose tissue undergo hypertrophic apoptosis, accompanied by substantial cell death, which triggers a strong stress response in the immune system and induces chronic inflammation in the body [4,5,6]. Although a direct causal relationship between inflammation and type 2 diabetes has yet to be clearly established, increasing evidence suggests that chronic inflammation may be one of the key drivers in the onset and progression of diabetes.

Adipose tissue, as the largest energy storage organ in the human body, plays a crucial role in regulating energy metabolism and maintaining energy balance. However, in the context of obesity, dysfunction of adipose tissue leads to systemic metabolic disturbances, which can subsequently trigger chronic inflammation and insulin resistance (IR). The phosphatidylinositol-3-kinase (PI3K)/protein kinase B (AKT) signaling pathway is a key intracellular signaling pathway that transmits various critical cellular signals and is involved in processes such as cell proliferation, differentiation, metabolism, and apoptosis [7,8,9]. The PI3K/AKT signaling pathway plays a central role in insulin signaling by promoting glucose uptake and utilization, thereby maintaining glucose homeostasis. Additionally, the PI3K/AKT signaling pathway directly influences insulin sensitivity by inhibiting apoptosis and promoting cell survival. Therefore, abnormal activation or inhibition of the PI3K/AKT signaling pathway may represent a significant mechanism underlying obesity-related metabolic diseases. Toll-like receptors (TLRs) are pattern recognition receptors that recognize pathogen-associated molecular patterns and are widely expressed on the surface of immune cells. Activation of TLRs can initiate various signaling pathways, leading to the expression of inflammatory cytokines and mediating innate immune responses [10,11,12]. Recent research revealed that TLRs play not only a critical role in antimicrobial defense but also a significant role in the development and progression of chronic inflammation and metabolic diseases. In particular, during obesity-associated chronic low-grade inflammation, TLRs exacerbate IR and accelerate the onset of type 2 diabetes by activating signaling pathways such as nuclear factor κB (NF-κB), which promotes the production of inflammatory cytokines. NF-κB is a transcription factor widely present in eukaryotic cells, initially discovered in B lymphocytes. NF-κB regulates the expression of various genes associated with immune responses and inflammation by specifically binding to regulatory elements in target genes [13,14,15]. It plays a central role in cellular stress, immune responses, inflammation, and cell survival. The PI3K/AKT signaling pathway acts as an upstream regulator of NF-κB, modulating its activity through multiple mechanisms. For instance, the PI3K/AKT pathway can indirectly enhance NF-κB activation by inhibiting the degradation of NF-κB inhibitor proteins (IκB), thereby promoting NF-κB nuclear translocation and activation, which exacerbates inflammation and IR. Khatua et al. reported that PI3K-mediated NF-κB pathway activation induces the expression of anti-apoptotic proteins, further driving the persistence and chronicity of inflammatory responses [16].

Based on the aforementioned background, this study aimed to construct an obese rat model to investigate the expression changes of the PI3K/AKT and TLR/NF-κB signaling pathways under obesity and their roles in obesity-related metabolic diseases. By employing intraperitoneal glucose tolerance tests (IPGTT), intraperitoneal insulin tolerance tests (IPITT), hematoxylin and eosin (HE) staining, and Western blot analysis, this study systematically examined the expression changes of proteins associated with the PI3K/AKT and TLR/NF-κB signaling pathways in the obese rat model and explored their relationship with IR and chronic inflammation. The results were expected to provide new theoretical insights into the mechanisms of obesity-related metabolic diseases and offer references for developing targeted therapeutic strategies.

## 2 Materials and methodologies

### 2.1 Experimental animals

This study utilized 200 8-week-old specific pathogen-free male Wistar rats (weight 180–200 g), purchased from Beijing Luhe Hospital Affiliated to Capital Medical University. The rats were housed in standard conditions prior to the experiment. They were acclimated for 7 days in an environment with a relative humidity of 55 ± 5%, a constant temperature of approximately 25°C, and a 12-h light/dark cycle, with a standard diet and *ad libitum* water. After the acclimation period, rats exhibiting normal activity were selected for subsequent experiments. Throughout the study, strict adherence to the “3Rs” principle – reduction, reuse, and recycling – was maintained to minimize the number of animals used and to alleviate pain while ensuring research objectives were met. Additionally, all experimental procedures followed animal welfare and humane care guidelines.


**Ethical approval:** The research related to animal use has been complied with all the relevant national regulations and institutional policies for the care and use of animals. The research protocol was approved by the local Ethics Committee (Approval No: 202062), which confirmed the scientific validity and ethical compliance of the study.

### 2.2 Construction and grouping of obesity rat models

This study established an obese rat model with IR induced by a high-fat diet (5.4 kcal/g, with fat content up to 46.5%). At the start of the experiment, all rats were randomly assigned to the control group (Ctrl group, *n* = 40) and the observation group (Obs group, *n* = 160). The Ctrl group was fed a standard diet (3.8 kcal/g, fat content 10%), while the Obs group received a high-fat diet. After 8 weeks of feeding, the rats’ body weight and anogenital distance were measured, and Lee’s index (Lee’s index = (body weight × 1,000)^1/3^/anogenital distance) was calculated to assess the degree of obesity. For the rats in the Obs group, insulin sensitivity was measured using the high-insulin euglycemic clamp technique, and rats meeting the obesity criteria based on Lee’s index were selected. Rats not meeting the obesity criteria were excluded from the study. Ultimately, a model of obese rats with IR was successfully established.

In the Obs group, the remaining rats were randomly divided into four subgroups: the model group, the PI3K/AKT inhibition group, the TLR/NF-κB inhibition group, and the combined PI3K/AKT + TLR/NF-κB inhibition group, with 40 rats in each subgroup. The animal models were constructed as shown in Table 1.

**Table 1:** Construction of animal models

Groups		Number of rats	Treatment for rats
Obs group	Ctrl group	40	Normal rats fed with ordinary diet
	Model group	40	Simple obese rats
	PI3K/AKT inhibition group	40	Obesity rats with abnormal glucose metabolism + PI3K/AKT inhibitor LY294002 injected via tail vein
	TLR/NF-κB inhibition group	40	Obesity rats with abnormal glucose metabolism + injection of TLR/NF-κB inhibitor BAY11-7082 via tail vein
	PI3K/AKT + TLR/NF-κB inhibition group	40	Obesity rats with abnormal glucose metabolism + injections of BAY11-7082 and LY294002 via tail vein

### 2.3 Observation indicators

(1) General conditions, including body weight, anogenital distance, and 24-h food intake of the rats, were measured before the intervention (week 0) and at weeks 2, 4, 6, and 8 of the intervention. Lee’s index was then calculated.
(2) The IPGTT was conducted at week 6 of the intervention. Rats were fasted for 14 h prior to the test. Fasting blood glucose was first measured (recorded as 0 min). Subsequently, a 50% glucose solution was intraperitoneally injected based on the rats’ body weight, and blood glucose levels were assessed at 15-, 30-, 60-, and 120-min post-injection.
(3) The IPITT was also performed at week 6 of the intervention. Rats were fasted for 14 h before the test, and fasting insulin levels were first measured (0 min). Insulin was then intraperitoneally injected based on body weight, with insulin levels measured at 15-, 30-, 60-, and 120-min post-injection.
(4) Glucose infusion rate (GIR) was measured. At the end of the intervention, 10 rats from each group were randomly selected to undergo a high-insulin euglycemic clamp procedure to assess insulin sensitivity.

### 2.4 Weight determination of adipose tissue and skeletal muscle and HE staining

After serum samples were collected, the rats were euthanized, and white adipose tissue from the abdominal reproductive organs and perirenal fat was extracted from each group. The tissue was blotted dry with filter paper and weighed using an electronic balance with an accuracy of 0.1 mg. Each sample was weighed three times, and the average weight was recorded.

A small piece of white adipose tissue from the same abdominal location was taken from each group of rats, fixed in 2.5% paraformaldehyde in ethanol, and subjected to gradient alcohol dehydration before being embedded in paraffin. Sections were then stained with HE and examined for tissue morphology under a 400× optical microscope.

### 2.5 Western blot analysis

Western blotting was used to detect the expression of proteins related to the PI3K/AKT signaling pathway (including PI3K, phosphorylated PI3K [p-PI3K], AKT, and phosphorylated AKT [p-AKT]) and the TLR/NF-κB signaling pathway (including p-P65, TLR-4, and NF-κB).

First, adipose tissue from the rats was lysed on ice using pre-chilled RIPA lysis buffer, followed by centrifugation at 12,000 rpm for 15 min at 4°C. Protein concentrations were determined using the BCA method, and 10 μL from each sample was subjected to sodium dodecyl sulfate-polyacrylamide gel electrophoresis (SDS-PAGE). The SDS-PAGE gel was placed into the electrophoresis chamber, where an initial voltage of 80 V was set using an electrophoresis apparatus (Nippon Genetics, Germany). When the bromophenol blue marker reached the separating gel, the voltage was increased to 160 V. Upon completion of the electrophoresis, the stacking gel was removed, and the separating gel was assembled in the following order: sponge/filter paper/separating gel/polyvinylidene fluoride membrane/filter paper/sponge, and then placed into the transfer chamber. A wet transfer system (Bio-Rad Laboratories, Inc., USA) was utilized to transfer the proteins at 100 V for 60 to 120 min.

After transfer, the membrane was placed at 25°C and blocked with 5% non-fat milk for 2 h. The membrane was then washed three times for 10 min each with Tris-buffered saline with Tween (TBST). The primary antibody, specific to the target protein, was added, and the membrane was incubated overnight (approximately 24 h) at 4°C. Following this, the membrane was washed again three times for 10 min each with TBST. Subsequently, the secondary antibody, which targets the primary antibody, was added and incubated for 1 h at 25°C with shaking. After incubation, the secondary antibody was discarded, and the membrane was washed three times for 10 min each with TBST. Once washing was complete, the membrane was removed, blotted with filter paper to remove excess liquid, and placed in a darkroom. Chemiluminescence reagent (purchased from Beijing ZhiJie FangYuan Technology Co., Ltd.) was evenly applied to the membrane to ensure thorough exposure. Finally, the membrane was placed on a development board and detected using a gel imaging system, with grayscale values analyzed using the *ImageJ* image analysis system.

### 2.6 Statistical methods

Data analysis was performed using *SPSS 23.0*. Measurement data that followed a normal distribution are presented as mean ± standard deviation (*x̄* ± *s*). Comparisons among multiple groups were conducted using a one-way analysis of variance, while comparisons between two groups were made using an independent samples *t*-test. Categorical data are expressed as percentages (%) and analyzed using the *χ*² test. All statistical tests were two-tailed, with *P* < 0.05 considered indicative of statistically significant differences.

## 3 Results

### 3.1 Establishment of obesity rat models

**Figure 1:**
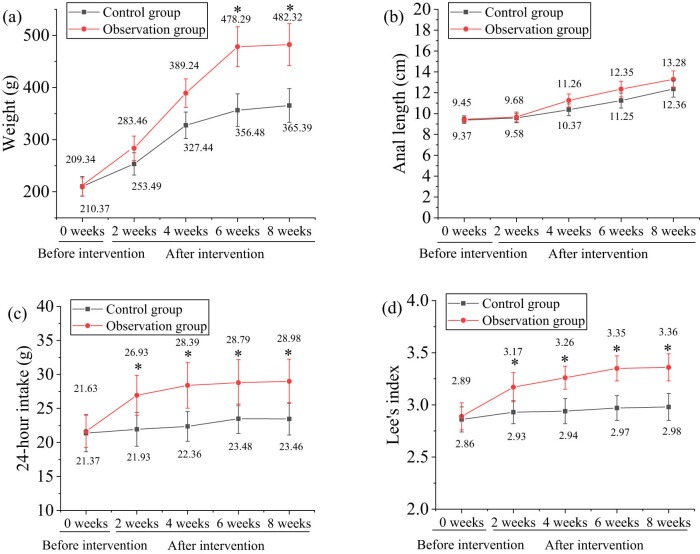
Comparison of body weight, anus-nose length, and 24-h food intake between the Ctrl and Obs groups. (a) Body weight; (b) anus-nose length; (c) 24-h food intake; and (d) Lee’s index. Note: *P < 0.05 vs Ctrl group.

Body weight, anogenital distance, and 24-h food intake of the rats were measured before the intervention (week 0) and at weeks 2, 4, 6, and 8 of the intervention, with results shown in Figure 1. At baseline, there were no significant differences among the groups in terms of body weight, anogenital distance, or 24-h food intake (*P* > 0.05). However, after 6 and 8 weeks of intervention, the body weight of rats in the Obs group was significantly higher than that of the Ctrl group, with statistical significance (*P* < 0.05). The 24-h food intake of the Obs group remained consistently higher than that of the Ctrl group at weeks 2, 4, 6, and 8, also showing statistical significance (*P* < 0.05). Additionally, the Lee’s index in the Obs group was significantly higher than that in the Ctrl group (*P* < 0.05), although there was no significant difference in anogenital distance between the two groups (*P* > 0.05). These results indicated that the high-fat diet successfully induced obesity in the rat model, achieving the desired experimental outcomes.

### 3.2 Results of IPGTT and IPITT

**Figure 2:**
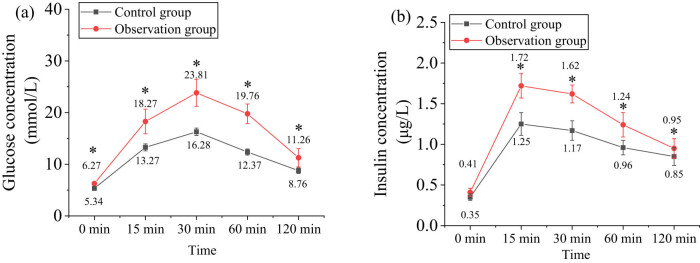
Results of IPGTT and IPITT. (a) IPGTT and (b) IPITT. Note: *marked considerable differences compared to the results of the Ctrl group (*P* < 0.05).

The statistical results for blood glucose and insulin levels at 0, 15, 30, 60, and 120 min during the IPGTT and IPITT tests are shown in Figure 2. The IPGTT results indicated that 30 min after intraperitoneal injection of 50% glucose, both Obs and Ctrl groups reached peak blood glucose levels. However, blood glucose levels in the Obs group were significantly higher than those in the Ctrl group, with statistical significance (*P* < 0.05). The IPITT results demonstrated that serum insulin levels peaked at 15 min after intraperitoneal insulin injection in both groups, with insulin levels in the Obs group significantly higher than those in the Ctrl group, also showing statistical significance (*P* < 0.05).

### 3.3 Contrast of GIR

**Figure 3:**
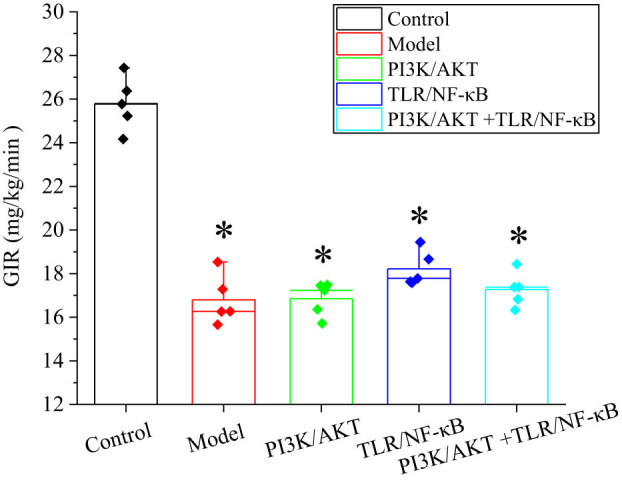
Comparison of GIR. Note: *indicated differences were substantial versus Ctrl group (*P* < 0.05).

In the comparison of GIR, rats in the model group, the PI3K/AKT inhibition group, the TLR/NF-κB inhibition group, and the PI3K/AKT + TLR/NF-κB combined inhibition group exhibited significantly lower GIR compared to the Ctrl group (*P* < 0.05). This indicates a marked reduction in insulin sensitivity among the rats in these treatment groups. Notably, the GIR in the model group was significantly lower than that in the Ctrl group, further validating the successful establishment of the obese rat model. These results clearly demonstrate the impact of different interventions on insulin sensitivity (Figure 3).

### 3.4 Weight determination of adipose tissue and skeletal muscle

**Figure 4:**
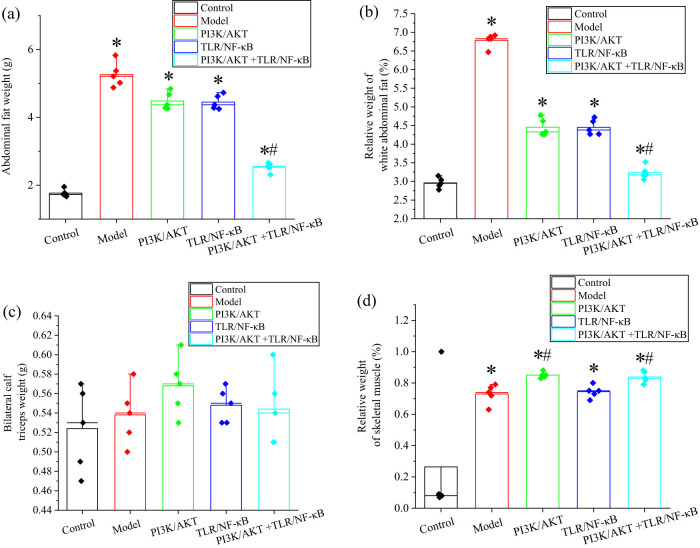
Results of adipose tissue weight and skeletal muscle weight. (a) abdominal fat weight; (b) relative weight of abdominal white fat; (c) weight bilateral triceps of calf; and (d) relative weight of skeletal muscle. Note: *and # indicated considerable differences compared to Ctrl group and model group, respectively (*P* < 0.05).

The results of the measurements for adipose tissue and skeletal muscle weight are shown in Figure 4. After 8 weeks of intervention, the abdominal fat weight and relative abdominal white fat weight in the Obs group were significantly higher than those in the Ctrl group, with statistical significance (*P* < 0.05). However, in the PI3K/AKT + TLR/NF-κB combined inhibition group, both abdominal fat weight and relative abdominal white fat weight were significantly lower compared to the model group, also showing statistical significance (*P* < 0.05).

Additionally, the relative skeletal muscle weight in all rats of the Obs group was significantly lower than that in the Ctrl group, with statistical significance (*P* < 0.05). Notably, the relative skeletal muscle weight in the PI3K/AKT inhibition group and the PI3K/AKT + TLR/NF-κB combined inhibition group was significantly higher than in the model group, also showing statistical significance (*P* < 0.05). However, there were no significant differences in the weight of the bilateral gastrocnemius muscles among the groups (*P* > 0.05). These results highlight the impact of different signaling pathway inhibitions on adipose tissue and skeletal muscle in rats.

### 3.5 HE staining of adipose tissue sections

**Figure 5:**
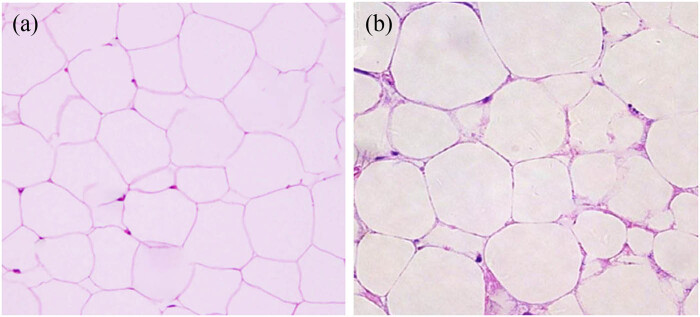
Morphological observation results of adipose tissues (×40 times). (a) Ctrl group and (b) Obs group.

Histological examination using HE staining revealed that the adipocytes in the Ctrl group rats were well-rounded, relatively small, uniform in size, and showed no signs of inflammatory cell infiltration. In contrast, the adipocytes in the Obs group rats were markedly enlarged, irregular in size, and appeared swollen. The fat droplets were enlarged, cell boundaries were indistinct, and there was evidence of cellular fusion (Figure 5). These HE staining results clearly demonstrate the impact of a high-fat diet on adipose tissue structure, further confirming the successful establishment of the obesity model.

### 3.6 Western blot analysis of related proteins in PI3K/AKT and TLR/NF-κB pathways

**Figure 6:**
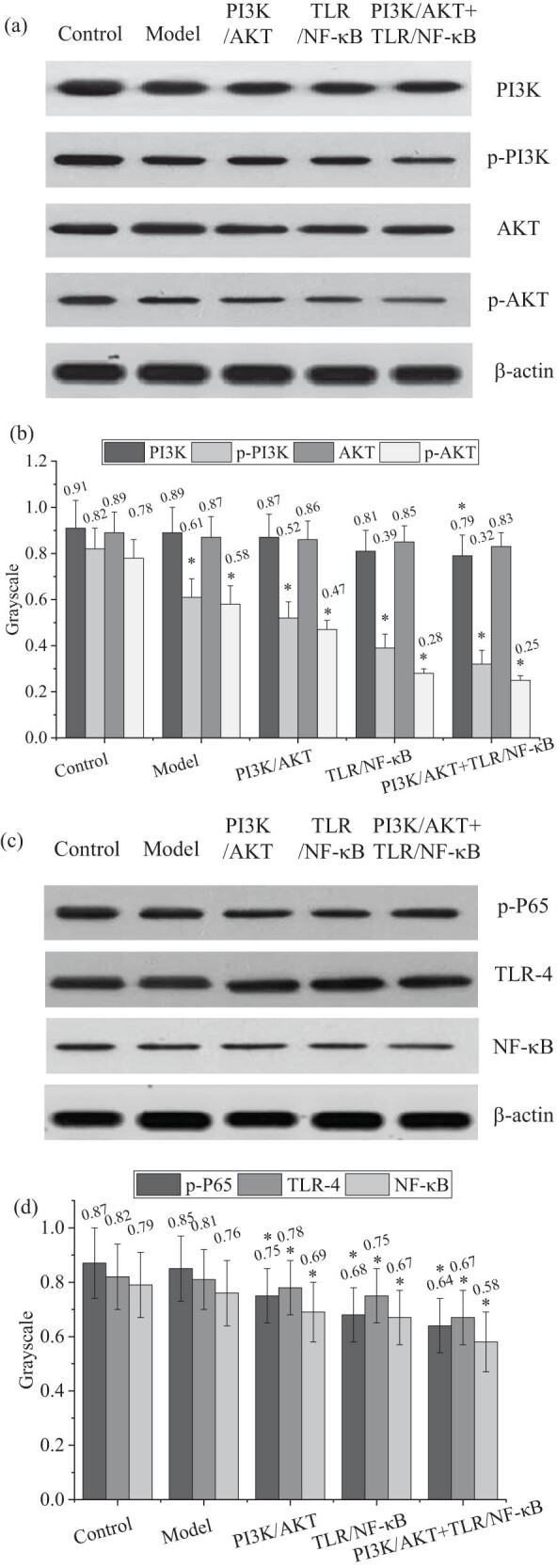
Expression levels of related proteins in PI3K/AKT and TLR/NF-κB pathways detected by Western blotting. (a) PI3K/AKT signaling-related protein levels; (b) PI3K/AKT signaling -related protein relative levels; (c) TLR/NF-κB signaling -related protein levels; and (d) TLR/NF-κB signaling-related protein relative levels. Note: *P < 0.05 vs Ctrl group.

By analyzing the signaling pathway proteins in adipose tissue, the expression of proteins related to the PI3K/AKT (including PI3K, p-PI3K, AKT, and p-AKT) and TLR/NF-κB (including p-P65, TLR-4, and NF-κB) signaling pathways across the different groups is shown in Figure 6. There was no significant difference in PI3K protein expression between the model and Ctrl groups (*P* > 0.05), but the expression of p-PI3K was significantly lower in the Obs group compared to the Ctrl group (*P* < 0.05). No significant difference in AKT protein expression was observed between the model and Ctrl groups (*P* > 0.05), while p-AKT expression was significantly reduced in the Obs group (*P* < 0.05).

Regarding the TLR/NF-κB pathway, there were no significant differences in p-P65, TLR-4, and NF-κB protein expression between the model and Ctrl groups (*P* > 0.05). However, in the PI3K/AKT inhibition group and the PI3K/AKT + TLR/NF-κB combined inhibition group, the expression of these proteins was significantly lower than in the Ctrl group (*P* < 0.05). These results indicated that inhibition of the PI3K/AKT and TLR/NF-κB pathways significantly reduced the expression of related proteins, thereby affecting IR and inflammatory responses.

## 4 Discussion

Obesity is a condition characterized by abnormal distribution or excessive accumulation of fat in the body. Simple obesity, which accounts for approximately 95% of all obesity cases, refers to obesity without underlying endocrine disorders. Changes in dietary habits, particularly excess food intake, are the primary causes of obesity. The nutritional obesity rat model is one of the most used animal models for obesity research, due to its many similarities with human obesity induced by high-fat diets, and is extensively used in studies on obesity and anti-obesity medications [17,18]. This study used a high-fat diet to simulate human obesity factors and induce obesity in rats. Body mass index (BMI) is commonly used to measure obesity in humans, while Lee’s index is frequently used to assess obesity in rats [19]. In this experiment, rat body weight, snout-to-anal length, and 24-h food intake were measured before intervention (week 0) and at weeks 2, 4, 6, and 8 post-intervention. The results indicated that, after 6 and 8 weeks of intervention, the body weight of rats in the Obs group was significantly higher than that of the Ctrl group (*P* < 0.05). Additionally, the 24-h food intake in the Obs group was consistently higher than that in the Ctrl group at weeks 2, 4, 6, and 8 (*P* < 0.05), and Lee’s index was also significantly higher in the Obs group compared to the Ctrl group (*P* < 0.05). Schütte et al. investigated whether maternal diabetes during pregnancy, without accompanying obesity, negatively affects the metabolic health of male offspring [20]. The study used a rat model where maternal type 2 diabetes was induced by insulin receptor inhibition, and this condition was maintained during pregnancy. The metabolic health of male offspring was then assessed. The study found that the weight gain and blood glucose levels of male offspring during puberty and adulthood were similar to those of the Ctrl group. After a high-fat diet, there were no significant differences in weight, fat mass, glucose, or insulin tolerance tests. This suggests that, in this model, isolated maternal hyperglycemia and hyperinsulinemia (without obesity) did not significantly impact the metabolic health of the offspring. It was hypothesized that other maternal metabolic parameters, such as obesity, may play a more crucial role in fetal metabolic programming. Compared to our study, both studies utilized rat models to investigate metabolic health and focused on the impact of high-fat diets on rats, with both involving long-term observations of body weight and metabolic parameters such as glucose tolerance.

The impact of carbohydrates on glucose metabolism remains controversial. Data indicate that increasing the proportion of high-carbohydrate foods can lead to higher energy intake and increased metabolic load on insulin secretion, which may adversely affect obese individuals. However, recent studies have highlighted that the type of carbohydrates consumed is closely related to glucose metabolism [21,22,23]. The high-insulin euglycemic clamp technique operates by continuously infusing a fixed amount of insulin or adjusting GIRs to disrupt the body’s endogenous glucose-insulin feedback mechanism, thereby maintaining blood glucose at baseline levels while keeping plasma levels of exogenous insulin relatively high. When GIR and metabolic rate stabilize at equilibrium, GIR represents the rate of glucose uptake by tissues such as muscle, fat, and liver. Thus, GIR serves as a sensitive indicator of insulin action on peripheral tissues. A higher GIR reflects greater insulin sensitivity. In this study, intraperitoneal glucose and insulin injections were used to mimic poor dietary habits in humans. Results showed that blood glucose and insulin levels in the Obs group were significantly higher (*P* < 0.05), while their GIR was markedly lower than that of the Ctrl group (*P* < 0.05). These findings are consistent with those of Crites et al. [24], indicating decreased insulin sensitivity, impaired insulin function, and IR in obese rats.

PI3K activates AKT and catalyzes its phosphorylation, which implies that when the PI3K/AKT signaling pathway is activated, the proportion of p-AKT increases. Conversely, when the PI3K/AKT signaling pathway is inhibited, the expression of p-AKT decreases. The results indicated that the expression levels of p-PI3K and p-AKT were significantly reduced in both the PI3K/AKT inhibition group and the PI3K/AKT + TLR/NF-κB combined inhibition group (*P* < 0.05), suggesting that the activity of the PI3K/AKT signaling pathway was suppressed in these groups. After the successful establishment of the obesity rat model, the relevant indicators of the signaling pathways in the rats’ adipose tissue were further examined. This study experimentally verified the direct association between changes in the activity of the PI3K/AKT signaling pathway and the expression of its downstream protein, p-AKT. The results demonstrated that inhibition of the PI3K/AKT pathway significantly reduced the phosphorylation levels of related proteins, further highlighting the pathway’s crucial role in metabolic regulation and the pathology of obesity. By assessing the indicators of these pathways in an obesity model, we can gain deeper insights into the relationship between obesity and signaling pathway dysregulation. The primary function of the PI3K/AKT signaling pathway in adipocytes is regulating insulin signaling, promoting glucose uptake, and facilitating lipogenesis. In the obesity model, a high-fat diet typically induces IR, leading to decreased activity of the PI3K/AKT pathway. Consequently, impaired insulin signaling in adipocytes may manifest as reduced glucose uptake and increased fat accumulation, aligning with the characteristics of IR. Additionally, the significant reduction in the expression levels of p-PI3K and p-AKT proteins indicates pathway suppression. The TLR/NF-κB signaling pathway primarily participates in immune and inflammatory responses, with TLR-4 capable of recognizing molecules such as saturated fatty acids, activating downstream NF-κB, and promoting the release of inflammatory factors like TNF-α and IL-6. In the obesity model, a high-fat diet can induce chronic low-grade inflammation by activating the TLR-4/NF-κB pathway, representing a crucial mechanism behind metabolic disorders associated with obesity. Consequently, in the TLR/NF-κB inhibition group, the inflammatory response in adipocytes was significantly suppressed, with reduced expression levels of TLR-4 and NF-κB-related proteins, thereby decreasing the production of inflammatory factors, which may help alleviate obesity-related chronic inflammation. The PI3K/AKT + TLR/NF-κB combined inhibition group simultaneously inhibited both insulin signaling and inflammatory pathways, theoretically allowing for more pronounced metabolic disturbances. The inhibition of the PI3K/AKT pathway may exacerbate IR, while TLR/NF-κB pathway inhibition reduces the inflammatory response. As a result, the expression levels of p-PI3K, p-AKT, TLR-4, and NF-κB were significantly diminished in the combined inhibition group. Adipocytes displayed impaired insulin signaling alongside a lower inflammatory response, suggesting that this group’s experiments may elucidate the complex balance between metabolic dysregulation and inflammation under the joint action of these two pathways.

Ghaiad et al. investigated the effects of the thiamine derivative, alpha-lipoic acid (Sulbutiamine), on streptozotocin-induced diabetic nephropathy [25]. The study assessed the impact of alpha-lipoic acid on renal function and related signaling pathways by successfully inducing a diabetic nephropathy model in rats. The results indicated that alpha-lipoic acid treatment reduced fasting blood glucose levels and improved renal function in diabetic rats. Furthermore, alpha-lipoic acid significantly decreased the levels of TLR-4, NF-κB, MDA, and PKC and suppressed the production of pro-inflammatory cytokines TNF-α and IL-1β, as well as the levels of TGF-β1. The study also demonstrated that alpha-lipoic acid alleviated tissue pathological changes associated with DN, indicating its renal protective effects through antioxidant, anti-inflammatory, and anti-fibrotic mechanisms. In comparison to this study, both research efforts found that specific interventions could inhibit the activity of the TLR-4/NF-κB signaling pathway. The mentioned study suggested that alpha-lipoic acid reduces the expression of TLR-4 and NF-κB, thereby exerting anti-inflammatory effects. Conversely, our study showed that the PI3K/AKT inhibition group and the combined PI3K/AKT + TLR/NF-κB inhibition group significantly suppressed the activity of the PI3K/AKT signaling pathway and inhibited the activation of the TLR-4/NF-κB pathway. Both studies highlight the role of intervention measures in reducing inflammation, demonstrating that modulation of the TLR-4/NF-κB and other related signaling pathways can play a crucial anti-inflammatory and protective role in metabolic disorders associated with diabetes or obesity.

## 5 Conclusion

This study successfully established an obesity rat model and analyzed the expression of the PI3K/AKT and TLR/NF-κB signaling pathways using methods such as IPGTT, IPITT, HE staining, and Western blotting. The results indicated that a high-fat diet activated the PI3K/AKT and TLR-4/NF-κB signaling pathways in adipocytes. This activation appears to be mediated by the regulation of protein levels, including PI3K, p-PI3K, AKT, p-AKT, p-P65, TLR-4, and NF-κB. A limitation of this study is that it assessed changes in body composition only through the relative weights of abdominal fat and skeletal muscle, lacking a more precise analysis of body composition. Future research should include detailed body composition analyses, contingent upon available resources and time. These findings provide a theoretical basis for further exploring the mechanisms underlying obesity-related metabolic diseases and may offer insights for the development of future research or clinical intervention strategies.

## References

[1] Li J, Wang T, Liu P, Yang F, Wang X, Zheng W, et al. Hesperetin ameliorates hepatic oxidative stress and inflammation via the PI3K/AKT-Nrf2-ARE pathway in oleic acid-induced HepG2 cells and a rat model of high-fat diet-induced NAFLD. Food Funct. 2021;12(9):3898–918. 10.1039/d0fo02736g. 33977953

[2] Ruze R, Liu T, Zou X, Song J, Chen Y, Xu R, et al. Obesity and type 2 diabetes mellitus: connections in epidemiology, pathogenesis, and treatments. Front Endocrinol (Lausanne). 2023;14:1161521. 10.3389/fendo.2023.1161521. PMC10161731 37152942

[3] Abbaszadeh F, Azizi S, Mobasseri M, Ebrahimi-Mameghani M. The effects of citrulline supplementation on meta-inflammation and insulin sensitivity in type 2 diabetes: a randomized, double-blind, placebo-controlled trial. Diabetol Metab Syndr. 2021;13(1):52. 10.1186/s13098-021-00669-w. PMC8097832 33952324

[4] Li H, Ren J, Li Y, Wu Q, Wei J. Oxidative stress: The nexus of obesity and cognitive dysfunction in diabetes. Front Endocrinol (Lausanne). 2023;14:1134025. 10.3389/fendo.2023.1134025. PMC10107409 37077347

[5] Abd El-Fattah EE, Saber S, Mourad AAE, El-Ahwany E, Amin NA, Cavalu S, et al. The dynamic interplay between AMPK/NFκB signaling and NLRP3 is a new therapeutic target in inflammation: Emerging role of dapagliflozin in overcoming lipopolysaccharide-mediated lung injury. Biomed Pharmacother. 2022;147:112628. 10.1016/j.biopha.2022.112628. 35032769

[6] Abd El-Hameed AM, Eskandrani AA, Salah Abdel-Reheim E, Abdel Moneim A, Addaleel W. The amelioration effect of antidiabetic agents on cytokine expression in patients with type 2 diabetes mellitus. Saudi Pharm J. 2024;32(5):102029. 10.1016/j.jsps.2024.102029. PMC10960149 38525262

[7] Bi H, Miao C. Effects of the combination of oestradiolvalerate and medroxyprogesterone acetate in repairing premature ovarian failure by regulating PI3K/AKT/MTOR signalling pathway-related proteins. Acta Med Med. 2022;4:2269.

[8] Abdollahi M, Marandi SM, Ghaedi K, Safaeinejad Z, Kazeminasab F, Shirkhani S, et al. Insulin-related liver pathways and the therapeutic effects of aerobic training, green coffee, and chlorogenic acid supplementation in prediabetic mice. Oxid Med Cell Longev. 2022;2022:5318245. 10.1155/2022/5318245. PMC9162863 35663196

[9] Wong CK, McLean BA, Baggio LL, Koehler JA, Hammoud R, Rittig N, et al. Central glucagon-like peptide 1 receptor activation inhibits Toll-like receptor agonist-induced inflammation. Cell Metab. 2024;36(1):130–43.e5. 10.1016/j.cmet.2023.11.009. 38113888

[10] Zhang Y, Liu J, Wang C, Liu J, Lu W. Toll-like receptors gene polymorphisms in autoimmune disease. Front Immunol. 2021;12:672346. 10.3389/fimmu.2021.672346. PMC8107678 33981318

[11] Zuo T, Yue Y, Wang X, Li H, Yan S. Luteolinrelieveddss-induced colitis in mice via HMGB1-TLR-NF-κB signaling pathway. Inflammation. 2021;44(2):570–9. 10.1007/s10753-020-01354-2. 33015735

[12] Jiang T, Li Y, He S, Huang N, Du M, Zhai Q, et al. Reprogramming astrocytic NDRG2/NF-κB/C3 signaling restores the diabetes-associated cognitive dysfunction. EBioMedicine. 2023;93:104653. 10.1016/j.ebiom.2023.104653. PMC10300300 37329577

[13] Chen X, Chen C, Fu X. Dendrobium officinale polysaccharide alleviates type 2 diabetes mellitus by restoring gut microbiota and repairing intestinal barrier via the LPS/TLR4/TRIF/NF-kB axis. J Agric Food Chem. 2023;71(31):11929–40. 10.1021/acs.jafc.3c02429. 37526282

[14] Fakhri S, Moradi SZ, Yarmohammadi A, Narimani F, Wallace CE, Bishayee A. Modulation of TLR/NF-κB/NLRP signaling by bioactive phytocompounds: a promising strategy to augment cancer chemotherapy and immunotherapy. Front Oncol. 2022;12:834072. 10.3389/fonc.2022.834072. PMC8921560 35299751

[15] Cao LP, Du JL, Jia R, Ding WD, Xu P, Yin GJ, et al. Effects of cyclophosphamide on antioxidative and immune functions of Nile tilapia (Oreochromisniloticus) via the TLR-NF-κB signaling pathway. Aquat Toxicol. 2021;239:105956. 10.1016/j.aquatox.2021.105956. 34496328

[16] Khatua S, Chandra S, Acharya K. Hot alkali-extracted antioxidative crude polysaccharide from a novel mushroom enhances immune response via TLR-mediated NF-κB activation: a strategy for full utilization of a neglected tribal food. J Food Biochem. 2021;45(1):e13594. 10.1111/jfbc.13594. 33346934

[17] Paone P, Suriano F, Jian C, Korpela K, Delzenne NM, Van Hul M, et al. Prebiotic oligofructose protects against high-fat diet-induced obesity by changing the gut microbiota, intestinal mucus production, glycosylation and secretion. Gut Microbes. 2022;14(1):2152307. 10.1080/19490976.2022.2152307. PMC9715274 36448728

[18] Martinelli I, Tayebati SK, Roy P, Micioni Di Bonaventura MV, Moruzzi M, Cifani C, et al. Obesity-related brain cholinergic system impairment in high-fat-diet-fed rats. Nutrients. 2022;14(6):1243. 10.3390/nu14061243. PMC8948807 35334899

[19] NgakouMukam J, Mvongo C, Nkoubat S, Fankem GO, Mfopa A, Noubissi PA, et al. Early-induced diabetic obese rat MACAPOS 2. BMC Endocr Disord. 2023;23(1):64. 10.1186/s12902-022-01252-8. PMC10026472 36935499

[20] Schütte T, Kedziora SM, Haase N, Herse F, Busjahn A, Birukov A, et al. Intrauterine exposure to diabetic milieu does not induce diabetes and obesity in male adulthood in a novel rat model. Hypertension. 2021;77(1):202–15. 10.1161/HYPERTENSIONAHA.120.16360. 33249866

[21] Miura A, Ikeda A, Abe M, Seo K, Watanabe T, Ozaki-Masuzawa Y, et al. Diallyltrisulfide prevents obesity and decreases miRNA-335 expression in adipose tissue in a diet-induced obesity rat model. Mol Nutr Food Res. 2021;65(14):e2001199. 10.1002/mnfr.202001199. 34014027

[22] Wu CC, Huang YW, Hou CY, Chen YT, Dong CD, Chen CW, et al. The anti-obesity effects of lemon fermented products in 3T3-L1 preadipocytes and in a rat model with high-calorie diet-induced obesity. Nutrients. 2021;13(8):2809. 10.3390/nu13082809. PMC8398352 34444969

[23] Molz P, Molz WA, Dallemole DR, Weber AF, Salvador M, Prá D, et al. Potential ameliorative effects of chromium supplementation on glucose metabolism, obesity, and genomic stability in prediabetic rat model. Biol Trace Elem Res. 2021;199(5):1893–9. 10.1007/s12011-020-02299-1. 32710349

[24] Crites S, Joumaa V, Rios JL, Sawatsky A, Hart DA, Reimer RA, et al. Moderate aerobic exercise, but not dietary prebiotic fibre, attenuates losses to mechanical property integrity of tail tendons in a rat model of diet-induced obesity. J Biomech. 2021;129:110798. 10.1016/j.jbiomech.2021.110798. 34700144

[25] Ghaiad HR, Ali SO, Al-Mokaddem AK, Abdelmonem M. Regulation of PKC/TLR-4/NF-kB signaling by sulbutiamine improves diabetic nephropathy in rats. Chem Biol Interact. 2023;381:110544. 10.1016/j.cbi.2023.110544. 37224990

